# Pharmacological and non-pharmacological therapies for chronic pancreatitis pain: a narrative review

**DOI:** 10.3389/fphys.2026.1857141

**Published:** 2026-06-22

**Authors:** Tareq Alsaleh, Mustafa Arain, John George

**Affiliations:** 1 Department of Internal Medicine, AdventHealth Orlando, Orlando, FL, United States; 2 Center for Interventional Endoscopy, AdventHealth Orlando, Orlando, FL, United States; 3 Pancreas Center, AdventHealth Orlando, Orlando, FL, United States; 4 Department of Gastroenterology and Hepatology, AdventHealth Orlando, Orlando, FL, United States

**Keywords:** chronic pancreatitis, endoscopy, pain, pain management, pancreas, surgery

## Abstract

**Importance:**

Chronic pancreatitis (CP) pain is the major driver of morbidity, reduced quality of life, healthcare use, and opioid exposure in affected patients. Pain in CP is heterogeneous and often correlates poorly with structural pancreatic abnormalities alone. It may arise from overlapping obstructive, neuropathic, nociplastic, and psychosocial mechanisms, making simple stepwise analgesic escalation insufficient for many patients.

**Observations:**

The emerging evidence supports a multidimensional strategy for treating CP pain. In some patients, pain is driven mainly by ductal obstruction, stones, strictures, inflammatory head masses, or other structural complications that may respond to endoscopic or surgical decompression. On the other hand, pain may persist because of pancreatic neuroplasticity, peripheral nerve injury, central sensitization, widespread hyperalgesia, and psychological distress. Newer tools such as the Comprehensive Pain Assessment Tool Short Form, electronic body mapping, and pancreatic quantitative sensory testing may help identify clinically relevant pain phenotypes beyond imaging alone. Although pharmacologic options remain limited, medications can provide relief in appropriately selected patients. Pregabalin has the strongest direct evidence among neuromodulators with favorable results. However, opioids remain widely prescribed even though they worsen dependency, opioid-induced hyperalgesia, and treatment resistance when used without reassessment of the dominant pain mechanism. Nonpharmacological modalities are an essential component. These include alcohol and smoking cessation, nutritional and endocrine optimization, and cognitive behavioral therapy. Endoscopic and surgical therapies are most effective when pain is anatomy-driven, while neuromodulation and other emerging interventions remain investigational but may be a promising option for nociplastic pain.

**Conclusions and relevance:**

Pain in CP should be approached as a dynamic biopsychosocial process by a multidisciplinary team. In addition to pain intensity, outcome measures should capture pain interference, function, quality of life, and opioid burden. Correctly identifying the pain phenotype and matching patients with the appropriate mechanism-based therapies and structural interventions will maximize treatment success and reduce prolonged opioid escalation and repeated low-yield interventions.

## 1 Introduction

Chronic pancreatitis is a fibro-inflammatory disorder of the exocrine pancreas that results in significant morbidity, with an incidence of 4.05 per 100,000 person-years and a prevalence of 41.76 per 100,000 persons in the United States (Yadav et al., 2011). Globally, the incidence is approximately 10 per 100,000 person-years, with the incidence roughly twice as high in men as in women (Xiao et al., 2016). The disease burden also has racial disparities, with Black patients more commonly having alcohol etiology, active tobacco smoking, longer disease duration, and higher pain and disability than White patients (Wilcox et al., 2016). Age at presentation varies by etiology, with hereditary or genetic forms often presenting earlier in life and alcohol- or tobacco-associated disease more commonly recognized later after prolonged environmental exposure. Early-onset, familial, idiopathic, or recurrent acute pancreatitis presentations should prompt consideration of hereditary predisposition and genetic counseling. The major risk factors and drivers of disease are alcohol and tobacco. Others include genetic mutations (PRSS1, SPINK1, CFTR, CTRC), pancreas divisum, and annular pancreas (Singh et al., 2019; Thierens et al., 2024).

Pain in CP is the major source of morbidity and decreased quality of life. It drives clinic visits, repeated admissions, and, over time, can come to dominate the illness itself. Pain occurs at some point in 84–90% of patients and affects not only physical function but also sleep, mood, and social well-being. Severe and constant pain, in particular, has been associated with substantially higher odds of depression, anxiety, sleep disturbance, and physical disability (Yadav et al., 2023; Thierens et al., 2024).

In clinical practice, the challenges associated with pain management in chronic pancreatitis include the unpredictable nature of pain symptoms and their poor correlation with structural disease. For example, two patients may have similar intraductal calcifications and associated pancreatic duct obstruction and yet report different pain symptoms ranging from being completely asymptomatic to severe, debilitating pain. Longitudinal data support that impression, as a nationwide cohort of 1,131 patients showed that neither pain severity nor pain character correlated reliably with morphologic findings, and pain patterns changed over time without clear relation to structural progression or disease duration (Kempeneers et al., 2021). Therefore, the traditional view of CP pain as a purely obstructive or inflammatory problem is no longer sufficient.

Ductal obstruction and tissue injury are still important in some patients, sometimes decisively so, but pain may also be maintained by peripheral nerve injury, pancreatic neuroplasticity, central sensitization, and nociplastic processes marked by widespread hyperalgesia and altered nociceptive processing (Saloman et al., 2023; Kuhlmann et al., 2026b). Psychological distress adds another important dimension. In a multicenter study, pancreatic duct obstruction, abnormal pain processing, and psychological burden each contributed independently to pain severity, and many patients had more than one of these drivers at the same time (Olesen et al., 2022).

Taking into consideration the multidimensional nature of CP pain, the limitations of a simple analgesic ladder become apparent. Furthermore, no randomized trials have evaluated first-line analgesics specifically in CP, and prolonged opioid escalation may worsen dependency, opioid-induced hyperalgesia, and overall treatment resistance. Therefore, in the real-world setting, management must be multidisciplinary, involving gastroenterology, pain medicine, endoscopy, surgery, endocrinology, nutrition, and behavioral health, and it should be guided by mechanism-based pain phenotyping rather than imaging morphology alone (Vege and Chari, 2022; Thierens et al., 2024).

However, management approaches often remain fragmented across analgesic therapy, behavioral care, endoscopic decompression, and surgery. This creates a practical gap for clinicians, particularly when repeated structure-directed interventions or empiric opioid escalation fail because neuropathic, nociplastic, or psychosocial mechanisms dominate (Singh et al., 2019; van Zeggeren et al., 2025). A clinically useful approach should begin with structured assessment of pain pattern, imaging morphology, opioid exposure, nutritional status, endocrine dysfunction, widespread pain, psychological distress, and sleep disturbance. This assessment allows treatment to be matched to the predominant phenotype: obstructive or nociceptive pain, mixed pain, or neuropathic/nociplastic pain, while universal supportive care is applied to all patients. This review addresses that gap by synthesizing pharmacological and nonpharmacological therapies for CP pain within a phenotype-guided framework, emphasizing mechanism-based treatment selection, opioid stewardship, multidisciplinary care, and timely escalation to endoscopic or surgical intervention when a structural driver is present.

## 2 Methods

This narrative review was developed through a targeted literature search of PubMed/MEDLINE and Google Scholar through February 2026. Search terms included combinations of “chronic pancreatitis,” “pain,” “abdominal pain,” “neuropathic pain,” “nociplastic pain,” “central sensitization,” “opioids,” “pregabalin,” “antioxidants,” “pancreatic enzyme replacement therapy,” “cognitive behavioral therapy,” “endoscopic therapy,” “surgery,” “celiac plexus block,” and “neuromodulation.” We prioritized peer-reviewed clinical trials, systematic reviews, meta-analyses, cohort studies, society guidelines, and mechanistic studies relevant to chronic pancreatitis pain mechanisms and treatment. Articles were selected based on clinical relevance, methodological quality, recency, and contribution to a phenotype-guided approach to pain management. Because this was a narrative review, formal systematic-review methods, risk-of-bias assessment, and quantitative synthesis were not performed. To improve interpretability, treatment options were assigned pragmatic evidence levels. High-level evidence was defined as consistent support from randomized trials, meta-analyses, or major society guidance in appropriately selected CP populations. Moderate-level evidence was defined as support from at least one randomized trial, multiple observational studies, or guideline-supported practice with some limitations in consistency, generalizability, or durability. Low-level evidence was defined as limited observational data, small studies, extrapolation from non-CP pain populations, or indirect support. Investigational or very-low-level evidence was assigned to therapies supported primarily by case series, pilot studies, early mechanistic data, or inconsistent sham-controlled results.

## 3 Clinically relevant mechanisms of chronic pancreatitis pain

Pain in CP almost never comes from a single source. In some patients, one mechanism clearly dominates, but more often, several drivers coexist. For practical purposes, four important domains are useful to recognize: obstructive or nociceptive pain, neuropathic pain, nociplastic pain, and psychosocial amplification.

### 3.1 Obstructive and nociceptive mechanisms

The most recognized mechanism involves pain arising from ductal or parenchymal disease. Pancreatic duct stones, fibro-inflammatory strictures, inflammatory head masses, and local complications such as pseudocysts can increase ductal pressure and sustain nociceptive input. Elevated intraductal pressure may also perpetuate fibrosis and parenchymal injury. Recent translational work using magnetic resonance cholangiopancreatography (MRCP) combined with computational fluid dynamics suggests that ductal pressure may eventually be estimated noninvasively, with encouraging concordance with invasive manometry and improvement after decompression (Zhao et al., 2026). Nevertheless, obstruction is only part of the picture, and endoscopic decompression does not reliably normalize pain, as randomized studies show favorable pain outcomes in a relatively small proportion of patients (Sheth et al., 2024).

### 3.2 Neuropathic mechanisms

Chronic inflammation reshapes pancreatic innervation through nerve hypertrophy, increased nerve density, neuritis, and upregulation of mediators such as nerve growth factor, TRPV1, and calcitonin gene-related peptide, supporting the concept of pancreatic neuroplasticity (Uc et al., 2021). These changes lower nociceptive thresholds and promote peripheral sensitization, resulting in pain that often persists after the original structural trigger has been treated. The morbidity associated with neuropathic pain was demonstrated in a real-world cohort of 681 patients with CP, where nearly one-third had mixed nociceptive and neuropathic pain features, and these patients reported worse quality of life than those with nociceptive pain alone (Saloman et al., 2023). In practice, these features correlate with patients whose imaging does not explain the severity of symptoms, yet whose pain remains persistent and functionally disabling.

### 3.3 Nociplastic mechanisms and central sensitization

Repeated nociceptive input may shift pain processing from the pancreas to the central nervous system, markedly altering the patient’s pain experience. Central sensitization can amplify pain signaling and produce widespread hyperalgesia, fatigue, and pain that appears disproportionate to pancreatic morphology. A recent study using electronic body maps illustrates this well: two-thirds of patients with CP pain reported widespread pain involving at least three body regions, and that phenotype was independently associated with higher pain severity, more neuropathic symptoms, fatigue, and worse physical and mental health (Machicado et al., 2026). Naturally, patients with central sensitization are often less responsive to interventions aimed only at structural disease, and prolonged opioid exposure may even worsen their problem through opioid-induced hyperalgesia (van Zeggeren et al., 2025).

### 3.4 Psychosocial amplification

Psychological distress is often treated as a secondary issue in CP, but it plays a critical role in the pain process. Depression, anxiety, sleep disturbance, pain catastrophizing, and ongoing substance use are common and independently worsen pain severity and treatment resistance. In a multicenter cohort of 201 patients, psychological distress had the strongest independent association with patient-reported burden, and more than half of patients had two or more overlapping pain drivers (Olesen et al., 2022). Therefore, routine screening for psychosocial comorbidity should be standard practice rather than an optional extension of care.

Chronic pancreatitis pain is best understood as a biopsychosocial phenomenon in which structural and nonstructural mechanisms interact (Vege and Chari, 2022). That framework is more clinically useful than any single-mechanism model, and it underpins the treatment decisions discussed below.

## 4 Pre-treatment assessment and pain phenotyping

Before treatment is escalated, the pain itself needs to be characterized properly. A useful assessment begins with the pattern of pain. Is it intermittent or constant? Meal-related or spontaneous? Relatively new, or present for years? Has it changed after previous endoscopic or surgical intervention? Current analgesic exposure is important as well, especially opioid use. Imaging remains essential, particularly when dilated ducts, stones, dominant strictures, inflammatory head masses, or small-duct disease are present, but imaging alone does not define the pain phenotype (Thierens et al., 2024).

Clinicians should also assess the temporal pain patterns over the disease course, where three broad patterns are observed. The first pattern, commonly referred to as Type A, consists of intermittent pain attacks separated by pain-free intervals. This may suggest episodic ductal obstruction, inflammatory flares, stone passage, pseudocyst-related symptoms, or recurrent acute-on-chronic pancreatitis. On the other hand, some patients experience persistent background pain with intermittent severe exacerbations, also referred to as Type B. These patients may have structural disease that generates nociceptive input mainly responsible for their baseline pain, while neuropathic sensitization or psychosocial distress amplify symptoms. Lastly, Type C manifests as chronic severe pain without clear severe attacks or pain-free intervals. This pattern may indicate advanced neuropathic or nociplastic pain, often with central sensitization, opioid-induced hyperalgesia, sleep disturbance, and psychological distress. This population often suffers from long-standing disease with reduced responsiveness to purely structure-directed interventions. Importantly, these patterns are not mutually exclusive and may evolve over time. Nevertheless, this classification is one tool that clinicians can use to decide whether management should prioritize decompression of obstructive disease, neuromodulatory therapy, behavioral interventions, opioid reduction, or multidisciplinary pain rehabilitation (Vege and Chari, 2022).

Other domains should be assessed in parallel. Exocrine pancreatic insufficiency, nutritional risk, and type 3c diabetes all influence symptom burden and treatment tolerance (Vege and Chari, 2022). Patient-reported outcomes are central tools for pre-treatment assessment. The PROMIS-29 and PROMIS Global Health instruments, validated in a real-world cohort, showed that severe and constant pain track closely with depression, anxiety, sleep disturbance, and physical disability (Yadav et al., 2023). Opioid exposure adds another layer of risk: about 44% of adults with definite CP are opioid users, and in a large propensity-matched analysis opioid use was independently associated with both acute-on-chronic pancreatitis and all-cause mortality (Kilani et al., 2025).

In day-to-day practice, a full multidimensional assessment can be difficult to perform consistently within the constraints of clinic schedules. That is where newer tools may be useful. The Comprehensive Pain Assessment Tool Short Form (COMPAT-SF) captures five important pain dimensions: severity, pattern, provocative factors, spread, and qualitative descriptors, and it has shown strong reliability and criterion validity (Kuhlmann et al., 2022). This score divides pain into three phenotypes: a low-burden phenotype, characterized by relatively mild pain across all dimensions, a high-intensity, constant pain phenotype, presenting as severe, persistent pain without significant fluctuation, and a widespread, multidimensional phenotype, where patients have pain that spreads beyond the pancreatic region with multiple qualitative descriptors (Kuhlmann et al., 2026b).

At the same time, an unavoidable limitation of most assessment tools is subjectivity, reporting, and measurement bias. More objective or semi-objective approaches are therefore needed. Electronic body mapping is one approach that identifies widespread pain patterns that correlate with neuropathic features, fatigue, and impaired function (Machicado et al., 2026). Another is pancreatic quantitative sensory testing (P-QST), which adds another level of granularity by separating patients into no, segmental, or widespread hyperalgesia phenotypes, with widespread hyperalgesia linked to constant pain and worse quality of life (Faghih et al., 2022). Importantly, 3-year follow-up data shows that P-QST phenotypes are dynamic, with 46% of patients changing phenotypes within the first year, highlighting the importance of continued follow-up and adjustment of pain control strategies for every patient (**Table 1**) (Kuhlmann et al., 2026a).

**Table 1:** Pain assessment and phenotyping tools in chronic pancreatitis.

Tool	Main domains assessed	Strengths	Limitations	Best clinical use	References
Numeric rating scale/visual analog scale	Pain intensity	Simple, rapid, widely used	Captures intensity only; does not assess pain mechanism, spread, function, or interference	Initial screening and longitudinal symptom tracking	(Kuhlmann et al., 2025)
Izbicki pain score	Pain frequency, intensity, analgesic use, work disability	Historically used in CP intervention trials	Less practical for routine clinic use; limited mechanistic detail	Comparing outcomes after endoscopic or surgical interventions	(van Veldhuisen et al., 2025a)
PROMIS-29/PROMIS Global Health	Pain interference, physical function, fatigue, sleep, anxiety, depression, social participation	Captures functional and psychosocial burden	Not specific to pancreatic pain mechanisms	Multidimensional assessment of disease burden and quality of life	(Yadav et al., 2023)
COMPAT-SF	Pain severity, pattern, triggers, spread, qualitative descriptors	CP-specific multidimensional pain tool; supports pain phenotyping	Still requires broader implementation and external validation in diverse clinical settings	Routine phenotyping beyond imaging findings alone	(Kuhlmann et al., 2022; Kuhlmann et al., 2026b)
Electronic body mapping	Pain location and spread	Identifies widespread pain patterns suggestive of nociplastic or centralized pain	Does not directly measure sensory thresholds or structural disease	Screening for widespread pain and central sensitization features	(Machicado et al., 2026)
Pancreatic quantitative sensory testing	Segmental and widespread hyperalgesia	Provides semi-objective sensory phenotyping	Requires specialized expertise and is not widely available	Research settings and specialized pain phenotyping	(Faghih et al., 2022; Kuhlmann et al., 2026a)

CP, Chronic pancreatitis.

**Figure 1:**
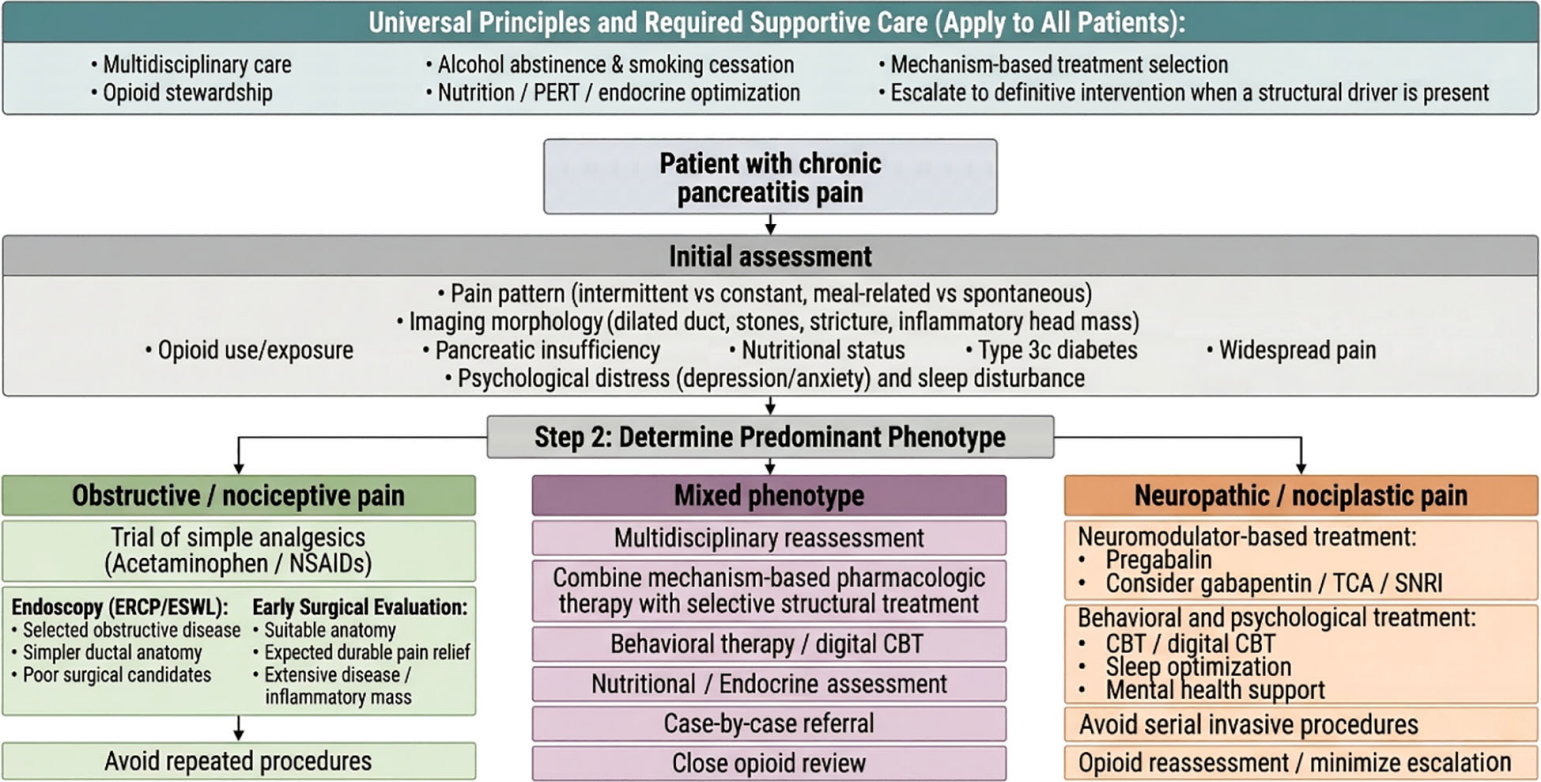
Mechanism-based treatment algorithm for chronic pancreatitis pain. Algorithm for managing chronic pancreatitis pain using phenotype-based care after initial assessment, with pathways for obstructive/nociceptive pain, mixed phenotype, and neuropathic/nociplastic pain.

Identifying the correct pain phenotype is a critical step before management. For example, patients with centrally sensitized or nociplastic pain are less likely to benefit from repeated peripheral interventions and more likely to be harmed by escalating opioid therapy (Kilani et al., 2025). Recognizing that phenotype early should shift the treatment plan toward neuromodulatory therapy and multidisciplinary management rather than serial procedures with little chance of durable success. This phenotype-based assessment provides the framework for treatment selection in chronic pancreatitis pain (**Figure 1**). Additionally, the identification of CP etiology plays an important role in patient assessment, as it can inform the clinician of modifiable drivers of ongoing injury, guide referral priorities, and clarify when structural intervention, addiction-medicine support, genetic counseling, or disease-specific therapy should be pursued (**Table 2**).

**Table 2:** Etiology-informed triage considerations in chronic pancreatitis pain.

CP etiology or dominant contributor	Pain-relevant considerations	Management priorities	References
Alcohol-associated CP	Ongoing alcohol exposure may perpetuate inflammation, oxidative stress, malnutrition, recurrent acute-on-chronic pancreatitis, and central sensitization	Alcohol abstinence support, addiction-medicine referral when appropriate, nutrition optimization, opioid stewardship, evaluation for obstructive complications	(Thierens et al., 2024; Göltl et al., 2024; Cohen et al., 2023)
Tobacco-associated CP	Smoking accelerates disease progression and is associated with greater pain burden and structural complications	Smoking cessation, relapse-prevention support, surveillance for disease progression, aggressive risk-factor modification	(Thierens et al., 2024; Göltl et al., 2024; Cohen et al., 2023)
Genetic or hereditary CP	Earlier onset, recurrent attacks, long disease duration, and higher lifetime intervention burden may occur	Genetic counseling when appropriate, early multidisciplinary referral, structural assessment, nutrition/endocrine monitoring, consideration of expert-center referral for refractory disease	(Singh et al., 2019; Thierens et al., 2024; Uc et al., 2016)
Obstructive or anatomic CP	Pancreas divisum, ductal strictures, stones, annular pancreas, or inflammatory head disease may produce ductal hypertension	MRCP/EUS-based structural assessment, ERCP/ESWL in selected patients, early surgical evaluation when durable obstruction is present	(Zhao et al., 2026; Sheth et al., 2024; Strand et al., 2022; Talukdar et al., 2024; van Veldhuisen et al., 2025a)
Autoimmune or active inflammatory etiologies	Pain may reflect active inflammation rather than fixed fibrosis alone	Confirm diagnosis, treat disease-specific inflammatory activity, avoid premature pain-procedure escalation if active inflammation is untreated	(Singh et al., 2019; Thierens et al., 2024; Cohen et al., 2023)
Idiopathic CP	Etiology may remain unclear despite evaluation; pain phenotype may be mixed	Complete etiologic workup, mechanism-based pain phenotyping, nutrition/endocrine assessment, individualized multidisciplinary care	(Thierens et al., 2024; Saloman et al., 2023; Cohen et al., 2023)

## 5 General principles of pharmacological management

Pharmacologic therapy in CP works best when it targets the dominant pain mechanism. Although this may be simple in some cases, it can be difficult to achieve in many patients, as a patient who initially has predominantly nociceptive pain related to inflammation or obstruction may later develop a mixed phenotype with neuropathic and centrally sensitized features (Kuhlmann et al., 2025). The World Health Organization pain ladder still offers a practical scaffold, but in CP it needs to be used with more judgment than in cancer pain (Thierens et al., 2024).

### 5.1 Acetaminophen and NSAIDs

Acetaminophen is thought to act through central inhibition of prostaglandin-mediated nociceptive signaling, with additional effects on descending serotonergic pathways (Leopoldino et al., 2019). NSAIDs inhibit cyclooxygenase enzymes and reduce peripheral prostaglandin synthesis, which may help when pain is driven by active inflammation or tissue injury (Brooks and Day, 1991). Both remain reasonable first-line options for mild to moderate nociceptive pain, especially earlier in the disease course, as these analgesics are familiar, inexpensive, and potentially opioid-sparing. The main shortcoming is the limited evidence base since no randomized controlled trials have evaluated these agents specifically in CP (Thierens et al., 2024). Their use is therefore extrapolated almost entirely from broader chronic pain practice, yet the CP population is not a generic chronic pain population. Malnutrition, renal vulnerability, and coexisting liver disease are common. Acetaminophen is often preferred when renal dysfunction, bleeding risk, or other contraindications limit NSAID use. NSAIDs may still help when inflammation is prominent, but they should be used cautiously (van Zeggeren et al., 2025). As pain becomes more neuropathic or nociplastic, the benefit of simple analgesics usually becomes less impressive, as their mechanistic impact on pancreatic neuropathy, central sensitization, or opioid-induced hyperalgesia is limited.

### 5.2 Neuromodulators

Neuromodulators target abnormal pain processing rather than pancreatic inflammation itself. Pregabalin and gabapentin bind the α2δ subunit of voltage-gated calcium channels, reducing excitatory neurotransmitter release from sensitized primary afferents (Taylor and Harris, 2020). In comparison, TCAs and SNRIs may act through descending inhibitory pain pathways by increasing synaptic norepinephrine and serotonin (Tao et al., 2019). These agents may dampen peripheral and central sensitization, potentially alleviating the burning, diffuse, sleep-disrupting pain that is often disproportionate to pancreatic morphology. However, their efficacy can be limited if anatomical causes, such as ductal obstruction, are driving the pain.

Among neuromodulators, pregabalin has the strongest direct evidence in CP. In a double-blind randomized trial, incremental pregabalin dosing over 12 weeks significantly improved Izbicki pain scores, quality of life, and daily functioning compared with placebo in patients without active inflammation or major ductal obstruction. Sedation and dizziness were common, but discontinuation rates were modest (Rana et al., 2025). A second randomized trial found that pregabalin combined with antioxidants reduced pain intensity, non-opioid analgesic use, and hospital admissions compared with placebo (Sureshkumar et al., 2021). A meta-analysis of randomized trials reached a similar conclusion, showing significant pain improvement with pregabalin relative to placebo (Khan et al., 2026).

The evidence is much thinner for gabapentin, tricyclic antidepressants, and serotonin-norepinephrine reuptake inhibitors. These agents do not have the same CP-specific trial base and are used largely by extrapolation from the neuropathic pain literature, where α2δ ligands, TCAs, and SNRIs remain standard first-line options (Soliman et al., 2025). Head-to-head analyses suggest no clear superiority between TCAs and gabapentinoids, while SNRIs and opioids may be associated with more adverse-event-related discontinuation (Sadegh et al., 2024). In practical terms, these agents tend to be most useful in patients whose pain persists despite control of structural disease, particularly when the pain is burning, shooting, diffuse, or highly disruptive to sleep (Cohen et al., 2021). Mood disturbance and insomnia may also help guide drug selection. This is one of the few areas in CP pain management where thoughtful clinical tailoring may matter as much as the formal evidence base.

### 5.3 Antioxidants

Antioxidants are intended to reduce oxidative stress and free radical injury. Therefore, the rationale for their use is biologically plausible: oxidative stress has long been implicated in pancreatic injury, and patients with CP may have relative depletion of antioxidant defenses (Grigsby et al., 2012). Even so, the trial literature has been inconsistent, and any analgesic signal appears modest at best, possibly because oxidative stress is only one component of CP pain, especially when established neuropathic remodeling or central sensitization become predominant mechanisms. That is why antioxidants are not considered standard therapy, as the American College of Gastroenterology has noted relative safety but uncertain biologic plausibility, lack of regulation, and poor standardization of dosing and formulation (Gardner et al., 2020).

Patient selection may be key when it comes to antioxidants. An RCT of 70 alcohol-induced chronic pancreatitis patients showed no benefit compared to placebo (Siriwardena et al., 2012). However, another RCT of patients, most of whom had idiopathic CP, experienced significant benefit with antioxidants (Bhardwaj et al., 2009). This may be because in alcoholic pancreatitis, antioxidants may not be able to overcome the oxidative stress that is continuously regenerated as long as alcohol exposure continues. This is further supported in pediatric prospective work, where alcohol is not the cause of CP. Antioxidant therapy produced a good pain response in 68% of children, although benefit was less frequent in those with advanced ductal disease (Gopan et al., 2023). Antioxidants may therefore have a role as adjunctive therapy, especially in cases not induced by ongoing environmental exposure, such as alcohol or smoking.

### 5.4 Pancreatic enzyme replacement therapy

Pancreatic enzyme replacement therapy is essential for exocrine pancreatic insufficiency. It improves fat absorption, reduces steatorrhea, supports nutritional status, and may lessen bloating or cramping related to maldigestion (Whitcomb et al., 2023). Furthermore, a proposed analgesic effect is the feedback inhibition of cholecystokinin release, which could reduce pancreatic stimulation and ductal pressure. However, this mechanism has not translated into consistent clinical benefit, likely because many patients have pain driven by fibrosis, ductal obstruction, neuropathic remodeling, or central sensitization rather than meal-stimulated pancreatic secretion alone. Reinforcing this, meta-analyses of randomized trials have not shown meaningful improvement in pain scores or analgesic use compared with placebo (Yaghoobi et al., 2016). Current guidance therefore recommends PERT for exocrine insufficiency, not for pain control (Gardner et al., 2020).

This distinction is easy to blur in practice because patients often experience subjective improvement after enzyme replacement. While that improvement is real and consistent, it usually reflects improved digestion and less postprandial discomfort rather than direct treatment of pancreatic pain (Vege and Chari, 2022).

### 5.5 Opioids and opioid stewardship

Opioids act primarily through μ-opioid receptor agonism in the central and peripheral nervous systems, reducing nociceptive transmission and altering pain perception. They should be reserved for severe pain that has not responded to more appropriate therapies. Even then, caution is not just advisable; it is necessary. Long-term opioid use may worsen central sensitization, dependency, constipation, and narcotic bowel syndrome, and it can precipitate opioid-induced hyperalgesia (Colvin et al., 2019; Thierens et al., 2024). A systematic review found that opioid-induced hyperalgesia may occur across a wide range of doses and often improves after opioid rotation, dose reduction, or the addition of agents such as ketamine or dexmedetomidine (Guichard et al., 2021). It is thus critical to avoid using opioids as a reflex response to persistent pain without renewed assessment of the underlying phenotype (van Zeggeren et al., 2025). Opioid stewardship in CP means more than limiting prescriptions, but also means using the lowest effective dose for the shortest feasible duration, favoring multimodal therapy, screening for misuse risk, monitoring functional benefit rather than pain scores alone, and involving pain specialists early when long-term therapy is being considered (Shah et al., 2021; Amakye et al., 2025).

### 5.6 Specialist-directed rescue approaches

Ketamine and intravenous lidocaine are increasingly discussed as rescue options for highly selected patients, particularly when opioid-induced hyperalgesia or refractory sensitization is suspected. Ketamine acts through N-methyl-D-aspartate (NMDA) receptor antagonism and may interrupt excitatory pain amplification. The National Institute of Diabetes and Digestive and Kidney Diseases (NIDDK) workshop participants noted that brief inpatient ketamine protocols can sometimes yield months of pain relief while reducing opioid requirements (Uc et al., 2021). In a recent cohort of patients undergoing pancreatectomy for CP, ketamine infusion provided opioid-sparing analgesia comparable to epidural therapy, with hallucinations as the most common adverse effect (Madduri et al., 2025). Intravenous lidocaine, which blocks voltage-gated sodium channels and may suppress ectopic discharges from injured or sensitized nerves, appears less consistently effective in practice. In a multicenter pilot study, mean pain reduction in CP was small overall, although nearly one-third of patients experienced clinically meaningful short-term improvement lasting up to one month (Augustinus et al., 2024). Importantly, these interventions belong in a monitored, multidisciplinary setting after standard approaches have failed.

Cannabinoids act through CB1 and CB2 receptors involved in nociception, inflammation, appetite, and affective pain processing, and although cannabis has shown some benefit in pain management in other populations (Wang et al., 2021), the data in CP is limited. No significant benefit was observed in an RCT of 24 patients (de Vries et al., 2016), while one observational cohort showed a promising decrease in mean daily opioid use (Barlowe et al., 2019).

Pharmacologic management in CP remains individualized. There is still no fully validated phenotype-guided prescribing algorithm, but the practical direction is reasonably clear: simple analgesics have a limited role, neuromodulators are more attractive when sensitization is suspected, exocrine insufficiency should be treated for digestive reasons rather than pain, and opioid exposure should be minimized whenever possible (Sheth et al., 2024; Kuhlmann et al., 2025; Lin et al., 2025). A mechanism-based summary of pharmacological therapies, their ideal candidates, and their main limitations is shown in **Table 3**.

**Table 3:** Pharmacological therapies for chronic pancreatitis pain.

Therapy	Primary target/mechanism	Best candidate phenotype	Expected role in practice	Main limitations	Suggested evidence level	Rationale	References
Acetaminophen/NSAIDs	Mild nociceptive pain; inflammatory pain	Early disease; intermittent pain; limited neuropathic/nociplastic features	Reasonable first-line trial for mild to moderate pain	No CP-specific randomized trials; limited efficacy in neuropathic/nociplastic pain; NSAID toxicity in selected patients	Low	No CP-specific RCTs	(Thierens et al., 2024; van Zeggeren et al., 2025; Kuhlmann et al., 2025; Brooks et al., 1991)
Pregabalin	Neuropathic pain; central sensitization	Persistent pain without major obstructive target; burning/shooting pain; sleep-disruptive pain	Best-supported neuromodulator in CP; useful opioid-sparing adjunct	Dizziness, somnolence; not all patients respond	Moderate	RCTs and Meta-Analysis support use	(Taylor et al., 2020; Rana et al., 2025; Sureshkumar et al., 2021; Khan et al., 2026)
Gabapentin/TCA/SNRI	Neuropathic pain modulation	Mixed neuropathic phenotype; pain persisting after structural causes have been addressed	Reasonable alternatives when pregabalin is ineffective or not tolerated	Evidence largely extrapolated from general neuropathic pain literature; adverse effects may limit use	Low	Limited CP-specific data	(Kuhlmann et al., 2025; Taylor et al., 2020; Tao et al., 2019; Soliman et al., 2025; Sadegh et al., 2024)
Antioxidants	Oxidative stress pathway (theoretical/adjunctive)	Selected patients with earlier disease; possible adjunct to neuromodulators	Optional adjunct in selected cases; not standard therapy	Inconsistent trial results; uncertain magnitude of benefit; formulation variability	Moderate	Multiple trials exist, but results are inconsistent and dependent on etiology	(Grigsby et al., 2012; Gardner et al., 2020; Siriwardena et al., 2012; Bhardwaj et al., 2009; Gopan et al., 2023)
Pancreatic enzyme replacement therapy (PERT)	Exocrine insufficiency; maldigestion-related symptoms	Patients with exocrine pancreatic insufficiency, steatorrhea, weight loss, or malnutrition	Essential for EPI and nutritional support; may improve overall symptom burden	Not a direct analgesic therapy for pancreatic pain	Low for pain control	RCTs do not support direct analgesic benefit	(Gardner et al., 2020; Whitcomb et al., 2023; Yaghoobi et al., 2016)
Opioids	Broad analgesia, not mechanism-specific	Severe refractory pain after better-targeted options have been tried	Rescue therapy only; short-term or highly selected use	Dependency, constipation, narcotic bowel syndrome, opioid-induced hyperalgesia, poor long-term fit for sensitized pain	Low for durable benefit but moderate evidence of harm	Long-term CP-specific benefit is poorly supported. High risk of opioid-induced hyperalgesia	(Kilani et al., 2025; Colvin et al., 2019; Guichard et al., 2021; Shah et al., 2021; Amakye et al., 2025)
Ketamine/IV lidocaine	Central sensitization; opioid-induced hyperalgesia; refractory pain	Highly selected patients with refractory pain, suspected sensitization, or opioid-related pain amplification	Specialist-directed rescue therapy in monitored settings	Limited CP-specific data; transient benefit in some patients; monitoring required	Low	Mostly pilot, perioperative, or specialist-center evidence	(Uc et al., 2021; Madduri et al., 2025; Wang et al., 2021)

NSAIDs, Non-steroidal anti-inflammatory drugs; CP, Chronic pancreatitis; RCT, Randomized controlled trial; TCA, Tricyclic antidepressants; SNRI, Serotonin norepinephrine reuptake inhibitors; EPI, exocrine pancreatic insufficiency; IV, Intravenous.

## 6 General principles of non-pharmacological management

Non-pharmacological care is often described as supportive, but that understates its importance. In many patients, it is the difference between pain that remains manageable and pain that becomes chronic, disabling, and opioid-dependent.

### 6.1 Alcohol abstinence and smoking cessation

Alcohol abstinence and smoking cessation are foundational because they address both disease progression and pain biology. In a cross-sectional study of 870 patients, former drinkers who maintained abstinence had less abdominal pain, less exocrine insufficiency, and fewer pseudocysts than those with a lifetime drinking history, and they were substantially more likely to remain relapse-free (Göltl et al., 2024). Prospective data also show that reducing tobacco and alcohol exposure independently predicts pain relief (Li et al., 2025).

The mechanistic rationale is explained by the pancreatic damage induced by alcohol and smoking. Both exposures accelerate inflammation, fibrosis, obstruction, and perineural injury, resulting in persistent nociceptive input that can foster central sensitization (Thierens et al., 2024). Smoking alone approximately doubles pain risk and is associated with more severe disease and greater need for surgery (Cohen and Kent, 2023; Göltl et al., 2024). Even without randomized pain-specific trials, the overall consistency of clinical and mechanistic data makes cessation support a first-line intervention for essentially all patients.

### 6.2 Nutritional optimization and exocrine care

Malnutrition is common in CP and often missed. Depending on the population studied, up to one-quarter of patients meet weight-based criteria for malnutrition, sarcopenia affects a much larger proportion, and micronutrient deficiencies are frequent (Cohen and Kent, 2023). These deficits matter clinically, because they worsen frailty, reduce resilience, impair neuropathic recovery, and amplify symptom burden, which in turn can blunt the effect of other pain interventions. In a large cross-sectional study, severe chronic pain correlated with lower levels of vitamin D, vitamin B12, folate, vitamin C, and magnesium (Goon et al., 2025). Routine dietitian involvement should therefore be a critical component of a multidisciplinary strategy. Nutritional counseling, assessment of oral intake, reinforcement of enzyme adherence, and targeted supplementation of documented deficiencies are all central components of care (Talukdar and Unnisa, 2022). This is also the setting in which PERT has its clearest value. By improving digestion and nutritional status, it can reduce the overall symptom load and improve quality of life even though it does not directly treat pancreatic pain (Vege and Chari, 2022). Furthermore, regular resistance exercise should be encouraged to reduce the risk of sarcopenia and osteopenia (Uc et al., 2016).

### 6.3 Endocrine optimization

Chronic inflammation and fibrosis can destroy pancreatic islet cells and lead to type 3c diabetes. Although diabetes is not itself a direct pain driver, poor glycemic control worsens catabolism, fatigue, sarcopenia, and overall functional decline, all of which can intensify symptom burden and complicate recovery (Vege and Chari, 2022). Endocrine optimization is therefore part of comprehensive pain care, especially in patients with progressive disease, weight loss, or fluctuating intake. Coordinated management with endocrinology often improves more than glucose control; it can also improve treatment tolerance and day-to-day functioning.

### 6.4 Psychological and behavioral therapies

Psychological and behavioral interventions have appropriately moved from the margins of CP care toward the center. Depression, anxiety, and sleep disturbance are highly prevalent and may independently worsen pain severity and interference (Patel et al., 2016; Fehér et al., 2026). Screening for these mental health issues, as well as disability, substance use, resilience, and social support should therefore be part of routine management rather than something added late in the course.

The most direct evidence comes from cognitive behavioral therapy. In the first randomized controlled trial of internet-based CBT for painful CP, participants assigned to CBT showed moderate to large improvements in pain intensity and pain interference at three months, and half achieved clinically meaningful pain reduction compared with only 13% of controls (Palermo et al., 2021). The ongoing IMPACT-2 trial is now testing this model at scale and should provide important effectiveness data (Palermo et al., 2026). Sleep is another critical component that is often overlooked. Poor sleep can reinforce central sensitization and weaken response to other therapies (Nijs et al., 2018; Ahmed et al., 2022). Physical activity may also help, although CP-specific randomized data remain limited. Exercise recommendations should therefore be individualized, particularly in patients with malnutrition, frailty, or brittle diabetes (Thierens et al., 2024).

## 7 Endoscopic therapies for chronic pancreatitis pain

Endoscopic therapy is most useful when pain is anatomy-driven. In practical terms, that means objective main pancreatic duct obstruction from stones or strictures, with symptoms that plausibly arise from ductal hypertension (Strand et al., 2022). It is not a general solution for all patients with CP pain, and outcomes are highly dependent on selection.

ERCP-based decompression remains the core approach. Small pancreatic duct stones in the head or neck may be removed after sphincterotomy using balloons or baskets, whereas larger radiopaque stones usually require extracorporeal shock wave lithotripsy followed by ERCP to achieve ductal clearance (Strand et al., 2022). Pancreatoscopy-guided lithotripsy is an alternative when ESWL is unavailable or unsuccessful, and early series suggest favorable technical outcomes, although durable comparative data remain limited (Sheth et al., 2025). Dominant main pancreatic duct strictures may respond to serial plastic stenting over several months, but recurrence after stent removal is common, and fully covered metal stents are not routinely recommended because of migration and *de novo* stricture risk. Symptomatic pseudocysts may also be managed endoscopically, although the degree of subsequent pain relief varies (Strand et al., 2022; Sheth et al., 2024). Durability is a key limitation, as endoscopic procedures often need to be repeated, and complete ductal clearance is difficult to achieve consistently. This is supported by findings from a recent trial comparing combined ERCP and ESWL to sham procedures, where pain relief was significant at 12 weeks but not 24 weeks (Talukdar et al., 2024).

Predicting response to endoscopic decompression can optimize patient selection. Secretin is a hormone that stimulates the exocrine pancreas to secrete bicarbonate-rich fluid into the pancreatic duct, increasing ductal pressure and causing temporary dilatation. Secretin-stimulated EUS showed a high (80-90%) accuracy for identifying obstructive pathology and predicting stent response in a prospective cohort from 1998. The principle is that ductal dilation after secretin stimulation indicates downstream obstruction (stricture, stone, sphincter dysfunction). A compliant duct that does not dilate suggests adequate outflow, whereas persistent dilation suggests obstruction that may benefit from stenting. This dynamic assessment of pancreatic duct compliance can theoretically help identify patients most likely to benefit from decompressive endotherapy (Catalano et al., 1998). However, no subsequent large-scale validation studies have been published, and this technique remains investigational rather than standard practice.

The long-term follow-up of the ESCAPE trial questioned the relative efficacy of endoscopic interventions, as early surgery produced lower Izbicki pain scores, more complete pain relief, and greater patient satisfaction than an endoscopy-first strategy. More than half of patients initially assigned to endoscopy ultimately required surgery, and delayed crossover was associated with worse outcomes (van Veldhuisen et al., 2025a). However, it is important to note that outcomes were similar between surgery and endoscopy in patients with early ductal clearance. Endoscopy only had worse results in patients who failed the initial intervention, requiring repeat procedures. This highlights endoscopy as a viable, minimally invasive option in high-volume expert centers where ductal clearance is more likely to be achieved on the first attempt (van Veldhuisen et al., 2025a).

Celiac plexus block is the best-studied procedural pain intervention in CP, but the effect is generally modest and short-lived. A meta-analysis of 11 studies found an overall efficacy of about 53% for EUS-guided celiac plexus block, with mean relief lasting roughly 81 days and a steep decline in responders over time (Machicado et al., 2025). EUS guidance appears superior to percutaneous techniques in randomized comparisons, but the evidence is low quality and no completed sham-controlled trial has definitively established benefit (Sheth et al., 2024; Wilcox et al., 2024). Adverse effects are usually transient, most often diarrhea and orthostatic hypotension, while major complications are rare (Wilcox et al., 2024; Machicado et al., 2025). Celiac plexus neurolysis is generally avoided in CP because durable benefit is poor and neuropathic pain may worsen (van Zeggeren et al., 2025).

## 8 Surgical therapies for chronic pancreatitis pain

Surgery has been traditionally considered a last-line measure, but that approach is changing as newer data support the shift toward earlier surgery in appropriately selected patients. The strongest argument is the long-term outcomes. Randomized trials consistently show that surgery provides better pain control than endoscopic therapy in obstructive CP (Boregowda et al., 2022; van Veldhuisen et al., 2025a). The previously mentioned long-term ESCAPE data is particularly compelling. Meta-analyses and recent guidance from ASGE and AGA now support early surgical evaluation in appropriate operative candidates with painful obstructive disease, reserving endoscopy mainly for patients who are poor surgical candidates or who strongly prefer a less invasive initial approach (Boregowda et al., 2022; Strand et al., 2022; Sheth et al., 2024). The patients most likely to benefit are those with clear structural disease: a dilated main pancreatic duct, ductal stones or strictures, or an enlarged inflammatory pancreatic head (Thierens et al., 2024). Patients with mixed or predominantly neuropathic or nociplastic pain can still undergo surgery, but outcomes are generally less favorable, especially after prolonged opioid use or many years of symptoms (Saloman et al., 2023; van Zeggeren et al., 2025). The ESCOPA multicenter study reinforced this point, showing that longer symptom duration and preoperative opioid exposure predicted less pain relief after surgery (van Veldhuisen et al., 2025b).

Procedure choice depends on morphology. Longitudinal pancreaticojejunostomy is best suited to diffuse ductal dilation with a relatively normal pancreatic head. When a dominant inflammatory head mass is present, duodenum-preserving pancreatic head resections such as the Frey or Beger procedure are usually preferred (Cohen and Kent, 2023). Randomized data and comparative meta-analysis suggest that these head-preserving operations provide strong pain relief while preserving function better than more radical resection (Ratnayake CBB. et al., 2020). Surgical drainage procedures have also shown excellent safety and pain outcomes in expert centers (van Veldhuisen et al., 2025c). Pancreaticoduodenectomy is generally reserved for suspected malignancy or inflammatory head disease too extensive for organ-preserving approaches, but it carries greater metabolic and nutritional consequences (Ratnayake CBB. et al., 2020). Distal pancreatectomy is used for disease localized to the body or tail, though pain outcomes are less predictable and fistula risk is meaningful (Lewellen et al., 2025). For diffuse, refractory disease, total pancreatectomy with islet autotransplantation has emerged as a specialized salvage option. In a prospective multicenter study, pain burden and opioid use declined substantially at one year, although insulin independence remained limited (Trikudanathan et al., 2025). This is not routine surgery. It requires careful multidisciplinary selection and should be concentrated in expert centers.

Surgical and endoscopic outcomes depend heavily on expertise and patient selection. A comprehensive pre-procedural evaluation should involve pancreatologists, pancreatic surgeons, radiologists, endoscopists, nutrition specialists, endocrinologists, and pain clinicians. In that setting, morbidity is lower, opioid discontinuation is more common, and pain relief is more durable (Ratnayake CBB. et al., 2020; Sheth et al., 2025; van Veldhuisen et al., 2025b). Key nonpharmacological, endoscopic, and surgical treatment options are summarized in **Table 4**.

**Table 4:** Nonpharmacological, endoscopic, and surgical therapies for chronic pancreatitis pain.

Therapy	Primary target/mechanism	Best candidate phenotype	Expected role in practice	Main limitations	Suggested evidence level	Rationale	References
Alcohol abstinence/smoking cessation	Disease modification; reduction in ongoing inflammatory and nociceptive drive	All patients, especially alcohol- or tobacco-exposed CP	Foundational therapy; should begin early	Benefit may be gradual; requires sustained behavioral support	Moderate	Supported by mechanistic rationale and observational data but randomized pain-specific trials are limited	(Göltl et al., 2024; Li et al., 2025; Cohen et al., 2023)
Nutrition/dietitian care/micronutrient replacement	Malnutrition, sarcopenia, vitamin deficiency, reduced treatment tolerance	Patients with weight loss, poor intake, frailty, or biochemical deficiencies	Core supportive care; improves resilience and overall symptom burden	Does not directly treat pancreatic pain; requires adherence and reassessment	Moderate	Strong supportive-care rationale and observational associations but limited pain-specific evidence	(Goon et al., 2025; Talukdar et al., 2022)
Endocrine optimization	Type 3c diabetes; catabolism; fatigue; metabolic instability	Patients with endocrine insufficiency or unstable glycemic control	Important supportive measure within comprehensive pain care	Indirect effect on pain; requires multidisciplinary coordination	Low	Important for metabolic stability and function but analgesic benefit is indirect	(Thierens et al., 2024; Vege et al., 2022; Cohen et al., 2023)
Behavioral therapy/CBT/digital CBT	Psychosocial amplification; pain coping; sleep and mood burden	Patients with pain interference, distress, maladaptive coping, or nociplastic features	Important adjunct; particularly valuable in mixed or centrally sensitized phenotypes	Access, adherence, and implementation remain barriers	Moderate	CP-specific randomized evidence is emerging. Relevant for centralized pain features	(Fehér et al., 2026; Palermo et al., 2021; Palermo et al., 2026)
Endoscopic decompression/ERCP/ESWL	Ductal hypertension from stones or strictures	Selected obstructive phenotype; simpler ductal anatomy; poor surgical candidates or less invasive preference	Structural therapy for selected patients with objective obstruction	Durability limited; repeat procedures common; less effective once sensitization dominates	Moderate	Supported in selected obstructive phenotypes but durability is variable and repeat procedures are common	(Sheth et al., 2024; Strand et al., 2022; Sheth et al., 2025; Talukdar et al., 2024)
Celiac plexus block	Visceral nociceptive pain modulation	Selected patients with predominantly visceral nociceptive pain who are poor candidates for other options	Temporary adjunctive option; not definitive therapy	Benefit modest and short-lived; limited usefulness in centrally sensitized pain	Low to moderate	Observational and meta-analytic evidence suggest short-term benefit, but durability is limited and evidence quality is variable	(van Zeggeren et al., 2025; Machicado et al., 2025; Wilcox et al., 2024)
Surgery	Durable relief of anatomy-driven pain; decompression/resection of structural disease	Dilated duct, obstructive disease, inflammatory head mass, favorable operative candidate	Most durable structural intervention; increasingly considered earlier in selected patients	Invasive; phenotype selection is critical; less effective in advanced centralized pain	High	RCTs and long-term data support superior durable pain relief in selected obstructive CP	(van Veldhuisen et al., 2025a; Boregowda et al., 2022; van Veldhuisen et al., 2025b; Ratnayake et al., 2020; van Veldhuisen et al., 2025c)
TPIAT	Removes pancreatic pain source while preserving islet function when possible	Diffuse, refractory disease in highly selected patients at expert centers	Specialized salvage option	Major procedure; endocrine consequences; limited insulin independence	Low to moderate	Prospective multicenter and expert-center data support benefit. No RCTs	(Trikudanathan et al., 2025)
Neuromodulation	Central pain modulation	Highly selected refractory patients, usually after standard options fail	Investigational or niche option	Evidence limited, heterogeneous, and not ready for routine use	Investigational	Small studies and mixed or negative sham-controlled results	(Ratnayake et al., 2020; Gulisano et al., 2024; Muthulingam et al., 2021; Andrade et al., 2024; Liu et al., 2024)

CP, Chronic pancreatitis; CBT, Cognitive behavioral therapy; ERCP, Endoscopic retrograde cholangiopancreatography; ESWL, Extracorporeal shockwave lithotripsy; RCT, Randomized controlled trial; TPIAT, Total pancreatectomy with islet autotransplantation.

## 9 Neuromodulation and other emerging interventions

Neuromodulation is an emerging area of real interest, but the evidence is still too limited for routine clinical use. Most of the literature consists of small studies, case series, or observational reports, which makes it difficult to know how much of the apparent benefit is durable and reproducible.

Virtual reality-based cognitive behavioral therapy (VR-CBT) shows promise for chronic back pain with moderate effect sizes, primarily through reducing catastrophizing and fear-avoidance (Li et al., 2024). While no direct evidence exists for chronic pancreatitis, the shared central sensitization mechanisms provide biological plausibility for extrapolation. However, chronic back pain is predominantly musculoskeletal/nociceptive with movement-related fear, whereas chronic pancreatitis involves visceral nociception with different neural pathways (Moreau et al., 2024).

Spinal cord stimulation has shown encouraging signals in uncontrolled cohorts, with reductions in pain and opioid use among successfully implanted patients. However, no significant benefit was observed in a recent sham-controlled trial and concerns remain regarding device-related complications, reintervention, and uncertain cost-effectiveness. For that reason, recommendations for spinal cord stimulation in CP remain premature (Ratnayake CB. et al., 2020; Gulisano et al., 2024).

Cervical vagus nerve stimulation has been tested in randomized sham-controlled crossover studies, but despite measurable changes in brain connectivity, it did not improve pain compared with sham (Muthulingam et al., 2021). Repetitive transcranial magnetic stimulation has produced a more promising signal, with sham-controlled trials reporting meaningful reductions in pain and opioid use, likely through endogenous opioidergic pathways. By contrast, transcranial direct current stimulation has not shown significant benefit (Andrade et al., 2024; Liu et al., 2024).

Other modalities remain early in development. Dorsal root ganglion stimulation may attract greater interest in the future based on broader chronic pain practice, but disease-specific evidence in CP remains extremely limited (Chapman et al., 2023). Scrambler therapy has favorable data in neuropathic pain more broadly but not yet in CP (Smith et al., 2023). For now, these interventions should be considered investigational and reserved for research settings or highly selected patients within an expert multidisciplinary program.

## 10 Future directions

The next major step forward in CP pain management will probably not come from a single new intervention. More likely, it will come from improved patient selection. Precision pain medicine is becoming less aspirational and more plausible. Recent multidimensional phenotyping work has already pushed the field in that direction. Cluster analysis using the COMPAT-SF identified clinically distinct pain phenotypes, including a widespread multidimensional phenotype associated with greater psychological distress and hyperalgesia, suggesting that treatment decisions should increasingly be based on mechanism rather than anatomy alone (Kuhlmann et al., 2026b).

Electronic body mapping offers a simple and scalable way to identify widespread pain and possible nociplastic features in routine practice, and it may help flag patients who are unlikely to benefit from repeated structure-directed interventions (Machicado et al., 2026). Pancreatic quantitative sensory testing adds another layer of sensory profiling and could, in time, help refine risk stratification and enrich future trials for patients with central sensitization (Faghih et al., 2022). Whether these tools will become practical outside specialized centers remains uncertain, but they are moving the field in an important direction.

Outcome measurement also needs to improve beyond simply measuring pain intensity alone, as it is too narrow for a disease that affects sleep, mood, physical function, nutrition, healthcare utilization, and opioid exposure. More meaningful endpoints will likely combine pain interference, functional status, quality of life, and treatment-related harms. The validation of CP-specific multidimensional tools and PROMIS-based frameworks gives future trials a stronger foundation than older single-score approaches (Kuhlmann et al., 2022; Yadav et al., 2023).

Digital therapeutics may also become increasingly relevant. The ongoing IMPACT-2 trial reflects a broader shift toward scalable behavioral care that can be embedded within real-world multidisciplinary models rather than limited to specialist centers (Palermo et al., 2026).

Several unanswered questions remain. Which patients with mixed nociceptive and nociplastic pain still derive meaningful benefit from endoscopic or surgical intervention? Can sensory profiling or biomarker-based approaches predict response to neuromodulators, surgery, or behavioral therapy? And how should future trials incorporate mechanism-based enrichment without becoming so selective that the results lose clinical relevance? Progress will likely depend on linking phenotyping, translational science, and pragmatic trial design more closely than has been done so far.

## 11 Conclusion

Pain in CP is heterogeneous, dynamic, and only partly explained by morphology. Managing it well requires more than stepwise analgesic escalation. It requires mechanism-based, phenotype-guided care that distinguishes obstructive pain from neuropathic and nociplastic pain while also addressing malnutrition, endocrine dysfunction, sleep disturbance, psychological distress, and substance exposure. Multidisciplinary care is central to that approach. So is timely escalation to definitive intervention when structural disease is clearly driving symptoms. In practice, two mistakes remain common: prolonged ineffective opioid escalation and delayed referral for endoscopic or surgical evaluation in appropriate patients. The field is moving, appropriately, toward matching the right therapy to the right pain phenotype earlier and with greater precision.

CP, Chronic pancreatitis; MRCP, Magnetic resonance cholangiopancreatography; EUS, Endoscopic ultrasound; ERCP, Endoscopic retrograde cholangiopancreatography; ESWL, Extracorporeal shockwave lithotripsy.
